# Remembering Emil von Behring: from Tetanus Treatment to Antibody Cooperation with Phagocytes

**DOI:** 10.1128/mBio.00117-17

**Published:** 2017-02-28

**Authors:** Stefan H. E. Kaufmann

**Affiliations:** Department of Immunology, Max Planck Institute for Infection Biology, Berlin, Germany

## Abstract

A century ago, Emil von Behring passed away. He was the first to be honored by the Nobel Prize for Medicine in 1901 for the successful therapy of diphtheria and tetanus, which he had developed from the bench to the bed. He also contributed to the foundation of immunology, since his therapy was based on passive immunization with specific antisera. Being an ambitious character, he did not shy away from friction with his colleagues Paul Ehrlich and Elias Metchnikoff and his mentor, Robert Koch. Behring was not only an excellent translational researcher but also a successful entrepreneur and early proponent of public-private partnerships.

**During our long-term studies about diphtheria (Behring) and tetanus (Kitasato), we also approached the questions of therapy and immunization, and for both infectious diseases we were able to cure infected animals as well as pretreat healthy ones so that they did not fall ill of diphtheria or tetanus.**

## EDITORIAL

With these words, Emil Behring (1854 to 1917) and Shibasaburo Kitasato (1853 to 1931) began their groundbreaking paper on diphtheria and tetanus immunity in experimental animals (1). The study demonstrated that sera from rabbits infected with *Clostridium tetani* conferred protection to naive mice against live tetanus bacilli and against tetanus toxin. In this paper, immunization against diphtheria was only briefly mentioned. One week later, Behring published a single-author paper on immunization of guinea pigs with inactivated *Corynebacterium diphtheriae* and diphtheria toxin and on protective activity of serum from immune animals (2). He concluded his paper stating that “The possibility of cures for even highly acute diseases can thus no longer be ignored.”

These findings aroused the attention not only of the medical community but also the public in general. Even though tetanus was a significant cause of neonatal and maternal death at the time, it was largely neglected and mostly feared as a killer of wounded soldiers. During the Franco-Prussian War (1870 to 1871) and the American Civil War (1861 to 1865), 350 tetanus cases with 90% mortality and 505 cases with 89% mortality were recorded, respectively. A cure for diphtheria, known at the time as the “strangling angel” of children, raised much higher attention as one of the most threatening killers, causing 1% of all deaths of children under the age of five years and with an overall childhood mortality of nearly 5% in Europe and the United States. In total, some 300,000 to 400,000 diphtheria cases were noted in the United States, England/Wales, France, and Germany, with half of the cases in Europe and half in the United States.

Behring started the first human trial of serum therapy against diphtheria in mid-January 1892; however, the test achieved limited success due to insufficient serum quality. It was only when larger animals were used and when Paul Ehrlich (1854 to 1915) developed standardization techniques that larger quantities of high-quality antidiphtheria serum became available for clinical trials.

In a 1894 report, the experimental results for 220 children suffering from diphtheria revealed an overall 77% cure, depending on the treatment initiation time (3). Treatment started on the first 2 days after diagnosis of disease was almost 100% successful, whereas by day 6, a steep decline to 50% was observed. Émile Roux (1853 to 1933)—who together with Alexandre Yersin (1863 to 1943) had characterized diphtheria toxin in 1888—was the first to use horses for immunization and reported a reduction from 50.7% fatal outcome of diphtheria without treatment to 24.5% with horse serum therapy. The first large animal harnessed for serum production by Behring and Ehrlich was a sheep provided by Robert Koch (1843 to 1910), who no longer needed the animal and wanted to save on the costs for its maintenance. However, soon they accepted horses as animals of choice for generation of high-titered diphtheria serum. Further refinement of the production and standardization of diphtheria antiserum resulted in a reduction of diphtheria mortality to 1 to 5% when given promptly after diagnosis. No wonder Behring was soon venerated as a “savior of children.” The first indications that serum therapy could cause adverse events also appeared, although Behring considered his serum innocuous. In 1896, a healthy infant was prophylactically treated with diphtheria serum because the housemaid had fallen ill of diphtheria. The child likely died of an anaphylactic reaction to the foreign serum, and in the obituary, the saddened father—a well-known pathologist—stated that this was “due to the injection of Behring’s serum for immunization” (15).

Early attempts to cure tetanus were rather disappointing. However, in 1895, Edmond Nocard (1850 to 1903) reported success with serum therapy in curing horses suffering from tetanus. Horses were of utmost economic importance at the time, for the transport of goods by breweries, dairy factories, garbage collection services, fire brigades, and the transport of passengers and military personnel. The anaerobic bacterium *C. tetani* flourishes in horse manure, and a light injury or routine neutering of an animal sufficed as a port of infection. After the outbreak of World War I (WWI), broad-scale serum therapy of wounded soldiers admitted to military hospitals was immediately introduced. This effort reduced the morbidity of soldiers with tetanus admitted to hospitals from >9 to <2 per 1,000 and reduced mortality to almost zero. As further illustration, at the beginning of WWI, 2,193 wounded soldiers were hospitalized in the military hospital in Namur, France, of whom 27 developed tetanus with 100% mortality. After the introduction of serum therapy, none of the 1,195 injured soldiers admitted in the following months developed tetanus. Similarly, after the introduction of serum therapy, tetanus cases among German, British, and U.S. armies dropped to virtually zero.

Following the enormous success of serum therapy, Behring turned his attention to research of intervention measures against the deadliest of all infectious diseases, tuberculosis (TB); however, his efforts were in vain. Strongly influenced by his successful experience with diphtheria and tetanus, Behring was convinced that pathology of TB could be deduced to a single toxin which he could then neutralize with an antiserum. As we know today, this was a far too simplified view, since TB is a highly complex disease in which both protection and pathogenesis are the outcome of a highly intertwined host-pathogen interplay. He abandoned his TB studies in 1908 without making any significant contribution. Then, following periods of frustration and depression, Behring published another breakthrough paper “About a new protective remedy against diphtheria” (4). A few weeks before the paper appeared, he reported his novel findings at the annual Congress for Internal Medicine, raising enormous interest not only in the medical arena but also in the public arena. The *Vossische* *Zeitung*, one of the major newspapers in those times, wrote that

**At today’s discussions, Behring appeared as lively as ever and reported on a new protective agent comprising a mixture of diphtheria toxin and anti-toxin. This agent was harnessed for treating individuals at risk prophylactically. It was found that first the agent was completely innocuous, second that the appearance of true protection could be demonstrated by the formation of sufficiently high abundance of protective agents in the blood of immunized individuals who all remained free of diphtheria (4).**

For years, Behring had attempted to inactivate diphtheria toxin without diminishing its immunogenicity, e.g., using heat, but without success. Ultimately, he succeeded by forming complexes of *a*ntitoxin plus *t*oxin, in a vaccine called diphtheria AT. Before Behring, Theobald Smith had reported the first successful vaccination of guinea pigs with antigen-antibody complexes (5). Broad-scale clinical testing of a preventive vaccine posed major strategic challenges. However, early tests performed soon after an outbreak showed that immunized children were consistently protected, whereas unvaccinated individuals in the affected area remained susceptible. Thus, in areas of five epidemics and in one area where diphtheria was endemic, full immunization of a total of 633 children resulted in only two diphtheria cases.

**FIG 1:**
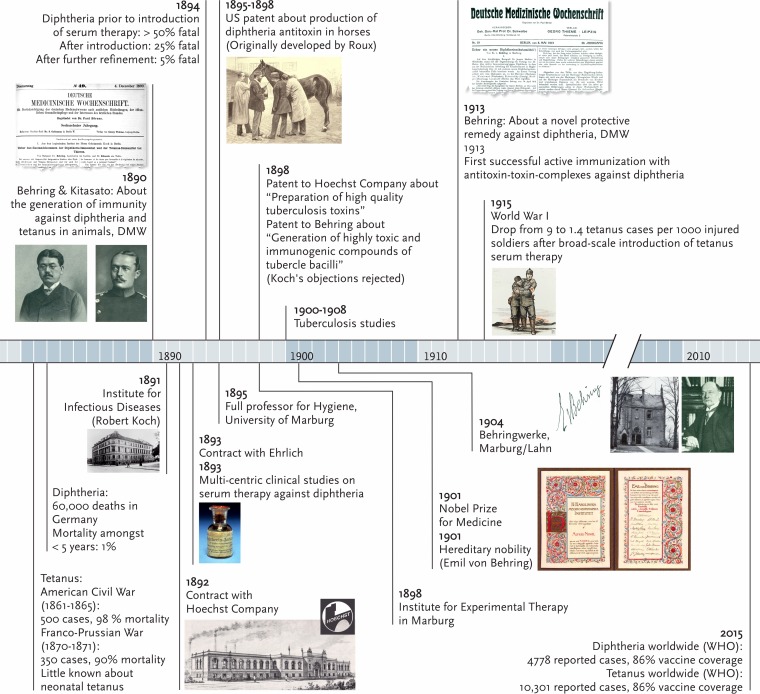
Timeline showing highlights of Emil Behring’s work as savior of children and wounded soldiers. DMW, Deutsche Medizinische Wochenschrift.

While Behring accomplished active vaccination against bacterial toxin producers, his efforts were developed further by the French researcher Gaston Ramon (1886 to 1963), in the 1920s (6). Ramon developed efficacious vaccines by inactivating diphtheria and tetanus toxins by means of formaldehyde, which allowed reproducible large-scale vaccine production at low cost. By adding an adjuvant, Ramon improved the efficacy of these vaccines (7). Aluminum hydroxide, the adjuvant used in today’s toxoid vaccines, was introduced by Glenny and colleagues (8). In 2015, with 86% vaccine coverage globally, according to the World Health Organization (WHO), cases of tetanus and diphtheria have dropped to 10,301 and 4,787 cases, respectively. See Fig. 1 for a timeline showing highlights of Behring’s work.

Emil Behring was born on 15 March 1854 in the district of Rosenberg in West Prussia, now Poland. He was the fifth of 13 children. His father was a poorly paid teacher unable to satisfy the higher education needs of his large family. When the opportunity arose to study medicine, tuition-free, during military service, Behring seized the opportunity. He received his military medical education at the Friedrich-Wilhelm Institute in Berlin, Germany, where research was an important part of the curriculum. Behring became interested in disease control and wound management by hygiene and antisepsis, following in the footsteps of Joseph Lister (1827 to 1912), the founder of antiseptic medicine. His first series of experiments dealt with chemical disinfection using iodoform. Behring considered iodoform not only antiseptic but also antitoxic, and he eventually turned to antitoxic serum activity. His first studies dealt with nonspecific antibacterial resistance of serum. These studies were performed at the Institute of Pharmacology of the University of Bonn, where he had been posted in 1888. In 1889, he was posted in Berlin, Germany, giving him the opportunity to work at the Hygiene Institute directed by Koch. It was at this vibrant research center that he shifted from nonspecific resistance to specific immunity against bacterial toxins. When the new Institute for Infectious Diseases was opened under the directorship of Koch, Behring joined together with many other outstanding researchers, including Kitasato and Ehrlich. He teamed up with Kitasato, who was experienced in the culture of anaerobic tetanus bacilli, to perform experiments that were published in the groundbreaking paper on toxin-destroying activity in serum. He also collaborated with Ehrlich, who developed methods for standardization of antisera against toxins, making this a first for a biological agent. Upon the recommendation of Koch, Behring concluded a lucrative contract with the company Lucius & Bruening in Hoechst (later called Hoechst Company) on the development and production of antitoxins for large-scale serum therapy of diphtheria and tetanus in 1892. Soon after, however, Behring and the Hoechst company sought the expertise of Ehrlich to reproduce standardized high-quality antiserum. Unfortunately, Behring and Ehrlich’s collaboration led to serious disputes regarding priorities and financial compensation.

When Behring failed to submit a patent on serum therapy and a French patent on the subject was submitted in Germany, both the Hoechst company and Behring objected successfully. Then, in 1895, Behring submitted a U.S. patent application for the generation of diphtheria antitoxin in horses, although it was Roux who first developed the method. Nevertheless, the patent was granted in 1898, without significant financial benefit for Behring. In the same year, both the Hoechst company and Behring (together with his coworker Wilhelm Ruppel) independently applied for patents on the generation of high-quality toxins from *Mycobacterium tuberculosis* for therapy of, and vaccination against, TB. Despite objections from Koch, who in 1890 discovered the source of the assumed tuberculous toxin—tuberculin, both groups were granted patents. This led to major confrontations between Behring and his mentor, Koch. In his wrangling, Behring fought relentlessly, later stating that “Regarding the interests of my person, I’ve only defended my intellectual property. This, however, with all ruthlessness at my disposal” (15). It took years before Behring reconciled with Koch and Ehrlich.

After the successful completion of clinical trials on serum therapy against diphtheria, Behring felt it was time to be a full professor of hygiene at a top German university. The first place he was considered for Professor of Hygiene was the University of Halle. His teaching activities, however, were meager, and he was rejected. Behring then insisted on being called full Professor of Hygiene at the University of Marburg, although another candidate had already been promoted. Again at the University of Marburg, the faculty did not receive Behring enthusiastically and rejected him. Nevertheless, in 1895, the ministry forced the university to accept Behring as a full professor and director of the Institute of Hygiene. During this unstable time, he decided to spend several weeks relaxing at the Mediterranean coast, where he repeatedly and urgently wrote the ministry requesting a professorship at a university where no local competition existed. In one letter to Friedrich Althoff, the Prussian Undersecretary of Education and Cultural Affairs, he promised: “If I can be accommodated in Marburg as a true full professor and director of the Hygiene Institute, then I hope to live in Germany as a calm and content citizen” (15). Despite his unstable situation, he coolly walked out of the Casino of Monte Carlo with winnings of 8,000 francs, enough for a sizable real estate purchase on the Isle of Capri.

Regardless of initial difficulties, Behring settled at the University of Marburg and became a respected faculty member. The year 1901 was the highlight of his professional and private life when he was the first scientist to ever receive the Nobel Prize for Physiology or Medicine, and he was honored with hereditary nobility, giving him the title Emil von Behring. In 1904, he founded the Behringwerke (Behring Company) as a spinoff of his academic work. The company produced a number of sera and vaccines and rapidly expanded when WWI broke out and the demand for vaccines, notably against tetanus, sky-rocketed. Behring died on 31 March 1917. The rescue of wounded soldiers from tetanus as well as increasing revenues from sales of vaccines were probably of little consolation in the face of the tragedy imposed by WWI.

Serum therapy of diphtheria and tetanus also contributed significantly to the foundation of immunology. Elias Metchnikoff (1845 to 1916) had shown in 1884 that specialized blood cells, which he termed fagocytes (now phagocytes), were able to engulf and eliminate bacteria and thereby founded the concept of nonspecific innate cellular immunity. Metchnikoff joined the Pasteur Institute in 1888, which rapidly became the center of innate cellular immunity. At Koch’s Institute for Infectious Diseases, Ehrlich became the proponent of specific acquired humoral immunity. By formulating the side chain theory, he provided the concept for antibody production and therefore also the scientific explanation for serum therapy. For years, the divergent concepts of these two schools led to intense discussions at international congresses and in publications, characterized by a lack of empathy and excess of bias. This created personal differences, not the least fueled by the often-aggressive tone of Behring. In retrospect, this divisiveness was viewed as symptomatic of Prussian-French animosity at the time. Behring, however, was equally abrasive toward Ehrlich, his colleague, and Koch, his mentor. In fact, his relationship with Metchnikoff was relatively friendly. Metchnikoff visited Behring twice, in 1891 with Roux, who was named godfather of one of Behring’s sons and in 1906 when Metchnikoff himself became godfather of another of Behring’s children.

Over the years, the two concepts of immunology began to merge. When complement was discovered by Jules Bordet (1870 to 1961), a nonspecific humoral component was identified. Soon complement was shown to serve as an effector of antibodies by Ehrlich, who also had coined the term. When Almroth Wright (1861 to 1947) described opsonization in the early 20th century, a mechanism by which antibodies facilitate bacterial phagocytosis, a further link between innate cellular immunity and acquired humoral immunity was obtained (9). Subsequently, the humoral and cellular immunology paths diverged again until the 1970s. Notably, the discovery of T lymphocytes as central players in macrophage activation fostered a dualistic thinking: the primary task of antibodies was seen in improved bacterial uptake by neutrophils (e.g., for encapsulated bacteria such as pneumococci) and that of T lymphocytes in macrophage activation (e.g., for tubercle bacilli). This dualism led to the concept of a distribution of responsibilities in antibacterial defense: antibodies and neutrophils being responsible for extracellular bacteria which are rapidly killed once phagocytosed and T lymphocytes and macrophages being responsible for intracellular bacteria which survive in resting macrophages but are controlled after appropriate activation. It is only now that we realize that such an oversimplification does not live up to the complexity of immunology. T lymphocytes also affect neutrophils, e.g., through interleukin 17 production, and antibodies affect the fate of intracellular bacteria in macrophages.

An early attempt to merge the two views and to improve personal relations are tangible in an equally humane and visionary letter from Metchnikoff to Behring on 29 November 1891.

**We can only support each other mutually, just as phagocytes and antitoxins do, since it can certainly be assumed that phagocytes receive major help from antitoxic activity, and that phagocytes, by capturing and destroying bacteria, provide major help for the host organism regarding its antitoxic defense (15).**

For further reading, see references [Bibr B10][Bibr B11][Bibr B15].
